# Factors affecting the accuracy of fetal cardiac ultrasound screening in the first trimester of pregnancy

**DOI:** 10.1007/s10396-024-01505-0

**Published:** 2024-11-01

**Authors:** Shin Hashiramoto, Mayumi Kaneko, Hiroko Takita, Yuka Yamashita, Ryu Matsuoka, Akihiko Sekizawa

**Affiliations:** Department of Obstetrics and Gynecology, Showa University Hospital, Showa University School of Medicine, 1-5-8 Hatanodai, Shinagawa-ku, Tokyo, 142-8666 Japan

**Keywords:** Congenital heart disease, First trimester, Ultrasound screening

## Abstract

**Purpose:**

Most studies on the performance of first-trimester cardiac screening have concentrated on comparing the detection rate between different protocols and not on the actual reason for false-negative results. Herein, we report the performance of first-trimester congenital heart disease (CHD) screening and factors that may affect the detection rate of CHDs.

**Methods:**

This retrospective observational study included patients who underwent first-trimester screening and subsequently gave birth at our facility. We analyzed the performance of first-trimester screening for CHD and major CHD (CHD requiring cardiac surgery or interventional catheterization within 12 months of birth).

**Results:**

Of the 6614 fetuses included, 53 had CHD and 35 had major CHD. For the prenatal diagnosis of CHD, the detection rate, specificity, positive predictive value, negative predictive value, and first-trimester detection rate for CHD were 64.1%, 99.9%, 94.4%, 99.7%, and 82.9%, respectively; the respective values for major CHD were 85.7%, 99.96%, 93.75%, 99.92%, and 85.7%. The detection rate was not significantly different when classified by crown-rump length or number of fetuses. A weak correlation was observed between low detection rate of major CHD and lower maternal body mass index (BMI) (correlation ratio: 0.17). The detection rate was significantly higher when the fetus was scanned with its spine at the 5–7 o’clock position (posterior spine) than at other positions (odds ratio: 3.82, 95% confidence interval: 1.16–12.5, *p* = 0.02).

**Conclusion:**

Posterior spine contributes to an improved diagnostic rate in first-trimester CHD screening. In addition, sonographers must recognize that low maternal BMI is a risk factor of false-negative results.

## Introduction

Congenital heart disease (CHD), the most common congenital malformation, is a major cause of neonatal mortality and morbidity worldwide [1]. Most CHDs occur in low-risk patients [2], indicating that universal screening is necessary. Heart development is considered to be completed by 8–10 weeks of gestation, allowing the detection of CHDs after 11 weeks of gestation [3]. Recent guidelines recommend screening for CHD during the first trimester of life [4]. Therefore, the timing of fetal CHD screening is being moved forward to the first trimester at many facilities.

Examination of the fetal heart and whole fetal body provides information on genetic abnormalities or syndromes the fetus may have, which provides an opportunity for accurate genetic testing and counseling. Early diagnosis provides parents with sufficient time to prepare for what they and their child may experience after birth. Particularly in regions where pregnancy termination has a gestational age limit, early diagnosis of critical malformations or particular genetic disorders has a tremendous impact on deciding whether to continue the pregnancy.

Over the last 30 years, the accuracy and limitations of first-trimester cardiac screening have been investigated in many studies using different protocols [4]. However, most studies have concentrated more on increasing the detection rate with different protocols and less on the actual reasons for false negatives. Because first-trimester cardiac screening requires a different skill set compared to second- and third-trimester evaluations, examiners who have never performed first-trimester cardiac screening may experience difficulties. Knowing the underlying factors for false-negative results has great significance in cases involving examiners at primary healthcare centers, where most patients are being screened. Herein, we discuss the accuracy of first-trimester CHD screening and the underlying factors affecting the accuracy of fetal cardiac ultrasound screening.

## Material and methods

### Study design and population

We conducted a retrospective observational study to elucidate: (1) the accuracy of the first-trimester screening protocol for CHD at our facility and (2) the underlying factors for false-negative results, possibly to identify a strategy to reduce them. We included consecutive patients who underwent first-trimester screening and had completed their perinatal period, up to delivery, at Showa University Hospital, a tertiary center in Tokyo, Japan, between April 2017 and May 2023. For each patient, the following variables were retrieved and analyzed: number of fetuses, maternal body mass index (BMI) at first-trimester screening, fetal crown-rump length (CRL) at first-trimester screening, gestational age at first-trimester screening, CHD type, karyotype, and pregnancy outcome. We classified CHD into two types: major and minor CHD. We defined major CHD as CHDs that required (or were expected to require) cardiac surgery or interventional catheterization within 12 months after birth and minor CHD as those that did not need (or were not expected to need) these interventions at all or at least not within 12 months after birth.

The study protocol was reviewed and approved by the Ethical Committee of Showa University (approval number M3389). Written informed consent was obtained from every participant.

### Outcome measurement

The diagnosis was confirmed via postnatal ultrasound, surgery, or catheterization for live births, a level-II scan conducted by a pediatric cardiologist during the second trimester for terminated pregnancies and intrauterine fetal demise (IUFD), or autopsy when informed consent was obtained. Normal neonates were discharged 5–6 days after birth and underwent routine follow-up by a pediatrician 1 month after birth.

We calculated the following parameters for CHDs and major CHDs: detection rate, specificity, positive predictive value, negative predictive value, and first-trimester detection rate in cases diagnosed prenatally with CHD. We surveyed factors that could explain the reason for the missed diagnosis, such as CRL, number of fetuses, maternal BMI, and position of the fetal spine. We defined the “posterior spine” position as a preserved image of the fetal heart with the spine at the 5–7 o’clock position.

### Ultrasound protocol

**Fig. 1:**
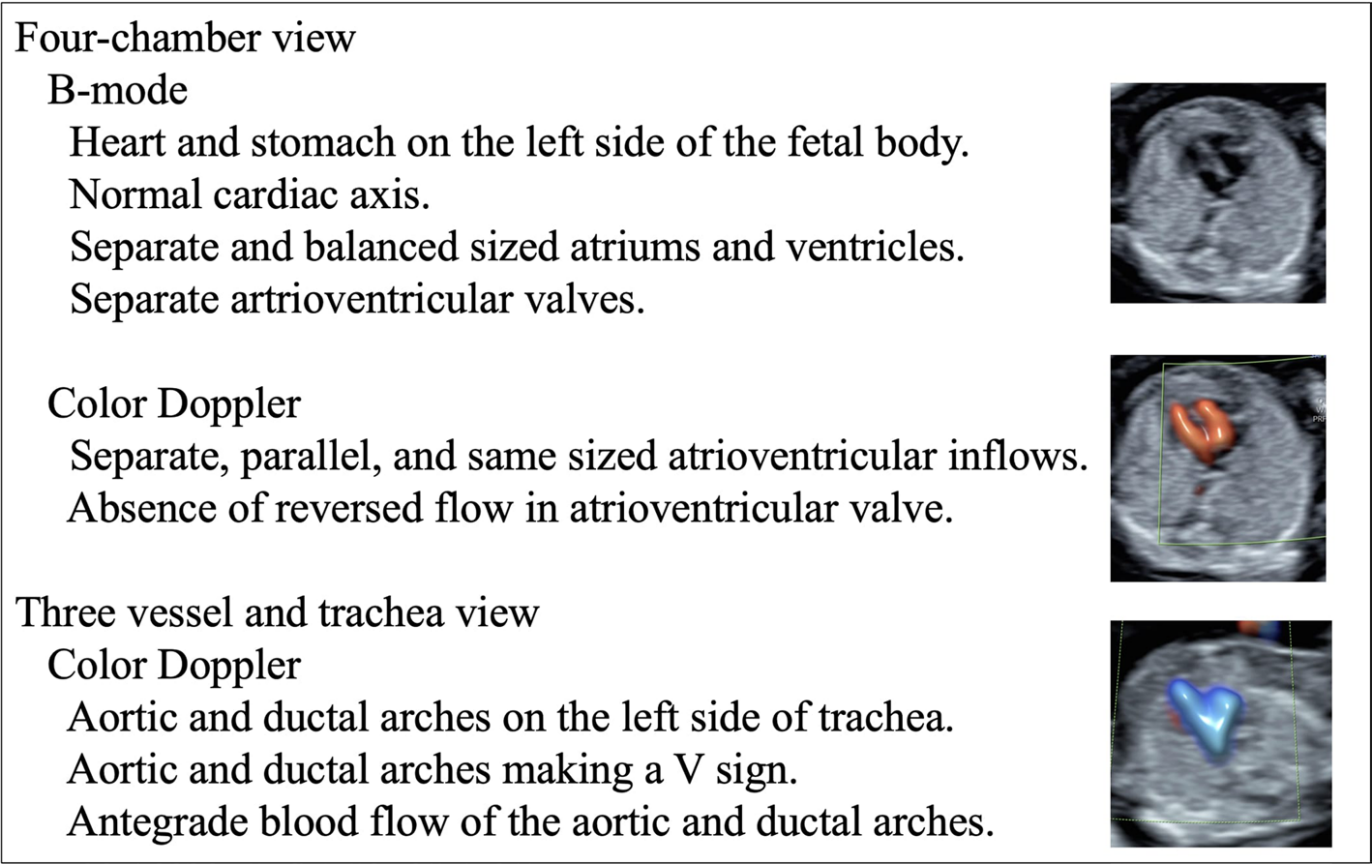
Checklist and images of fetal cardiac screening in the first trimester

We performed the first-trimester ultrasound screening between 11 + 0 and 13 + 6 weeks of gestation and when the fetal CRL was between 45 and 84 mm, as described in the Fetal Medicine Foundation protocol. Screened patients who did not match the appropriate time range or CRL were excluded from the study. We used a standardized protocol (Fig. 1) to screen for CHD. Board-certified fellows of the Japan Society of Ultrasonics in Medicine who have a license for Nuchal Translucency measurement certified by the Fetal Medicine Foundation confirmed the ultrasound images.

The operator attempted to evaluate the fetal heart in the apical view with the fetal spine in the posterior position. We took still images of the four-chamber view (4CV) and three-vessel and trachea view (3VTV), and a movie from the stomach to the top of the aortic arch in grayscale and color Doppler mode. When we could not obtain a satisfactory image for evaluation using transabdominal ultrasound, we used transvaginal ultrasound to obtain satisfactory images. The “as low as reasonably achievable” (ALARA) principle was strictly applied, especially when using the color Doppler mode, and we kept the soft tissue thermal index below 1.

Patients in whom fetal CHD was suspected following the first-trimester screening were referred to a pediatric cardiologist with over 20 years of experience in fetal medicine, and the type of CHD in the fetus was diagnosed.

The main ultrasound machine used during this period was Voluson E10 (GE Healthcare, Zipf, Austria), equipped with C1-5-D and RM6C transabdominal transducers and a RIC5-9A-RS transvaginal transducer.

### Statistical analysis

Group characteristics were examined using descriptive statistics. Frequency data were compared using the chi-squared test or Fisher’s exact test, as appropriate. The sensitivity, specificity, and positive and negative predictive values were calculated. Statistical analyses were performed using JMP data analysis software (JMP® Pro, Version 17.0.0; SAS Institute, Cary, NC).

## Results

### Study population

**Fig. 2:**
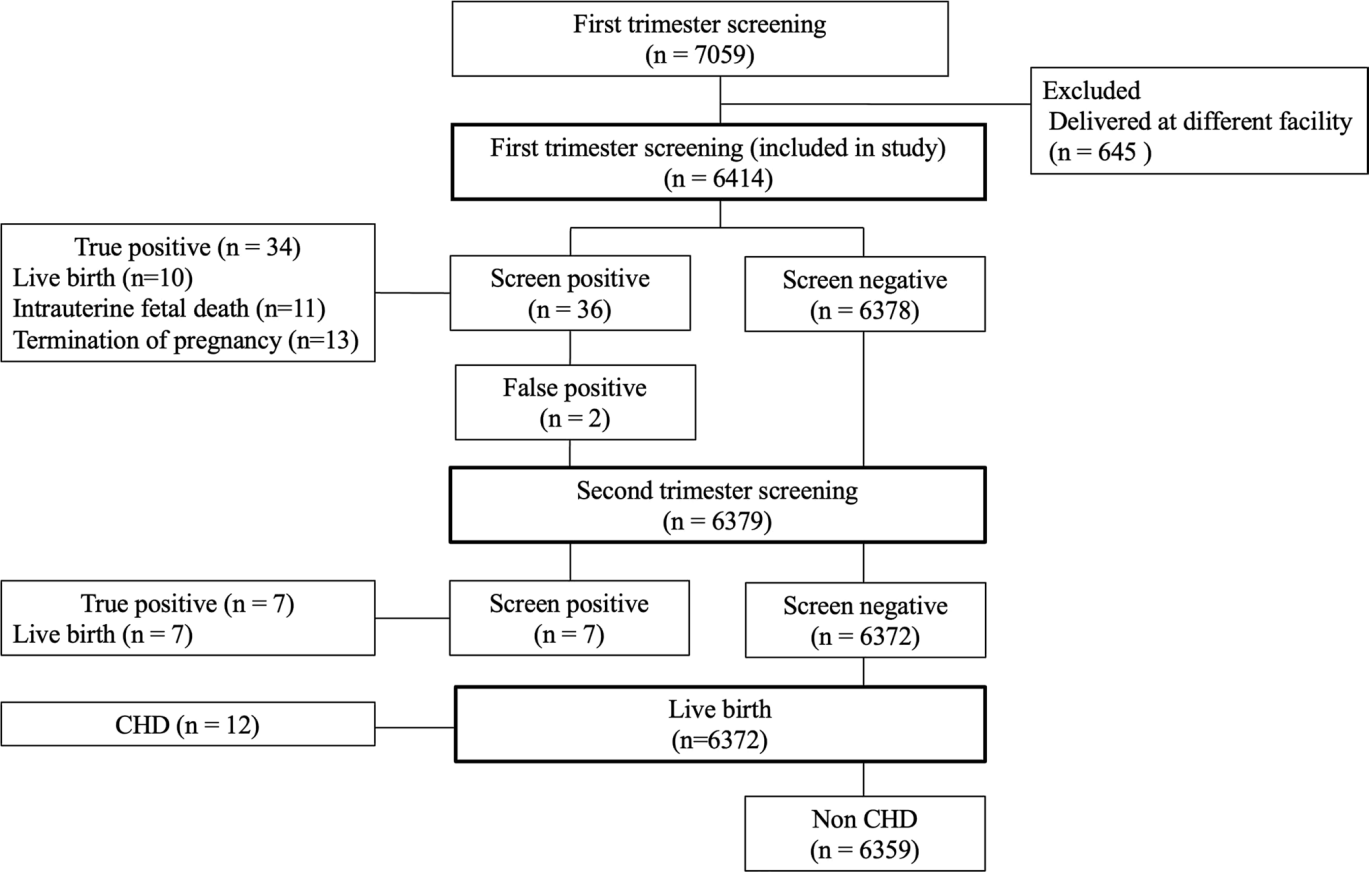
Flowchart summarizing the study population and congenital heart disease screening results

A total of 7059 fetuses underwent first-trimester screening in our facility. However, 645 fetuses were excluded because they were delivered at a different facility. The remaining 6614 fetuses were included in this study (Fig. 2). Maternal and fetal characteristics are shown in Table 1. The median maternal age, height, weight, and BMI were 35 years, 159.2 cm (range 138–181), 53.9 kg (range 34–116), and 20.5, respectively. The median CRL was 70.2 mm (range 45.4–84.0) and the gestational age at ultrasound was 13 + 2 weeks (range 11 + 0–13 + 6). There were 157 twin pregnancies and three triplet pregnancies.

**Table 1:** Maternal and fetal characteristics

	Median (range)
Maternal age (years)	34 (16–48)
Height (cm)	158.9 (131–180)
Body weight (kg)	52.6 (36–131)
BMI	20.2 (15.2–42.5)
CRL (mm)	66.7 (45.0–84.0)
Gestational week at ultrasound	13 + 0 (11 + 0–13 + 6)

*BMI* body mass index, *CRL* crown-rump length

### Performance of first-trimester CHD screening

Of the 6414 fetuses screened in the first trimester, 53 (0.82%) had CHD and 35 (0.54%) had major CHD. At the first-trimester screening, 36 fetuses were identified to have CHD, although two of them were ultimately diagnosed to have normal hearts (false positives). The abnormal findings at the first-trimester screening and the diagnosis are listed in Table 2. Of the 34 true-positive cases (including 30 major CHDs), 10 were live births, 11 were intrauterine fetal demise (IUFD), and 13 pregnancies were terminated (Fig. 2, Table 3). At the second-trimester screening, an additional seven fetuses were diagnosed with CHD (including five major CHD), and 12 additional fetuses were diagnosed with CHD after birth, including no major CHD (Table 3).

**Table 2:** True-positive congenital heart disease cases screened in the first trimester

Case	Type of ultrasound	Four-chamber view	Three-vessel trachea view	Diagnosis	Karyotype
1	Transabdominal	Unbalanced ventricles	No V sign	DORV	Normal
2	Transabdominal	Unbalanced ventricles	No V sign	DORV	Not performed
3	Transabdominal	Unbalanced ventricles	No V sign	DORV	Not performed
4	Transabdominal	Unbalanced ventricles	No V sign	DORV	Not performed
5	Transabdominal	Unbalanced ventricles	No V sign	DORV	Not performed
6	Transabdominal	Unbalanced ventricles	No V sign	DORV, PA	Not performed
7	Transabdominal	Unbalanced ventricles	No V sign	DORV	Trisomy 18
8	Transabdominal	Unbalanced ventricles	No V sign	DORV	Trisomy 18
9	Transabdominal	Unbalanced ventricles	No V sign	DORV	Trisomy 18
10	Transabdominal	Unbalanced ventricles	No V sign	HLHS	Trisomy 13
11	Transabdominal	Unbalanced ventricles	No V sign	HLHS	Trisomy 13
12	Transabdominal and transvaginal	Unbalanced ventricles	No V sign	HLHS	Trisomy 13
13	Transabdominal	Unbalanced ventricles	No V sign	TA, VSD	Not performed
14	Transabdominal	Unbalanced ventricles	No V sign	PA/IVS	Normal
15	Transabdominal	Unbalanced ventricles	No V sign	PA/IVS	Trisomy 18
16	Transabdominal	Unbalanced ventricles, butterfly sign	No V sign	Dextrocardia, DORV, AVSD	Normal
17	Transabdominal	Butterfly sign	Normal	AVSD	Trisomy 21
18	Transabdominal	Butterfly sign	Normal	AVSD	Trisomy 21
19	Transabdominal and transvaginal	Butterfly sign	Normal	AVSD	Not performed
20	Transabdominal	Butterfly sign	Normal	AVSD	Not performed
21	Transabdominal	Butterfly sign	Normal	AVSD	Trisomy 21
22	Transabdominal	Butterfly sign	No V sign	AVSD, TOF, PLSVC	Trisomy 21
23	Transabdominal	Butterfly sign	No V sign	AVSD, aberrant RCA, PLSVC	Trisomy 21
24	Transabdominal	Butterfly sign	Reversed aortic flow	AVSD、HLHS	Tetrasomy 9p
25	Transabdominal	Butterfly sign	No V sign	AVSD、HLHS	Trisomy 18
26	Transabdominal	Butterfly sign	No V sign	PA/VSD, parachute MV, PLSVC	Not performed
27	Transabdominal	Butterfly sign	No V sign	AVSD, TGA, DORV, PS	Not performed
28	Transabdominal	Left axis deviation	No V sign	DORV, TGA, VSD, CoA	Normal
29	Transabdominal	Left axis deviation	No V sign	VSD, MAPCA, PA	22q11.2 deletion
30	Transabdominal	VSD	No V sign	TA, VSD	Not performed
31	Transabdominal	VSD	Normal	VSD	Trisomy 18
32	Transabdominal	Normal	Right aortic arch	Right aortic arch, VSD	Not performed
33	Transabdominal	Normal	Right aortic arch	Right aortic arch	Not performed
34	Transabdominal and transvaginal	Right stomach	Normal	Heterotaxy, IVC absence	Not performed

*VSD* ventricle septal defect, *DORV* double outlet right ventricle, *PA* pulmonary artery atresia, *HLHS* hypoplastic left heart syndrome, *TA* tricuspid atresia, *PA/IVS* pulmonary atresia with intact ventricular septum, *AVSD* atrioventricular septal defect, *TOF* tetralogy of Fallot, *PLSVC* persistent left superior vena cava, *RCA* right coronary artery, *MV* mitral valve, *PS* pulmonary stenosis, *TGA* transposition of great arteries, *PS* pulmonary artery stenosis, *CoA* coarctation of aorta, *MAPCA* major aortopulmonary collateral artery, *RA* right atrium, *IVC* inferior vena cava

**Table 3:** Timing of detection and performance of first-trimester CHD screening

	Case	Timing of detection (%)			Performance of first-trimester CHD screening (95% CI)				
		First trimester	Second trimester	Postnatal	DR	Spec	PPV	NPV	DR in prenatally diagnosed CHD
CHD	54 (0.84)	34 (64.1)	7 (13.2)	12 (22.6)	64.1 (50.6–75.6)	99.9 (99.8–99.9)	94.4 (81.8–99.8)	99.7 (99.5–99.8)	82.9 (68.7–91.4)
Major CHD	35 (0.54)	30 (85.7)	5 (14.3)	0	85.7 (70.6–93.7)	99.9 (99.8–99.9)	93.7 (79.8–98.2)	99.9 (99.8–99.9)	85.7 (70.6–93.7)

*CHD* congenital heart disease, *DR* detection rate, *Spec* specificity, *PPV* positive predictive value, *NPV* negative predictive value, *CI*, confidence interval

The detection rate, specificity, positive predictive value, negative predictive value, and first-trimester detection rate for CHD were 64.1%, 99.9%, 94.4%, 99.7%, and 82.9%, respectively. The same values for major CHD were 85.7%, 99.96%, 93.75%, 99.92%, and 85.7%, respectively (Table 3).

### Details of false-negative cases

Details of the false-negative cases and possible reasons for missed diagnoses are listed in Table 4. Seven fetuses judged to have normal hearts at the first-trimester screening were diagnosed with CHD in the second trimester. Five patients had major CHD (cases a-e), and two had minor CHD (cases f and g). Twelve patients were diagnosed with CHD after birth; all cases were minor CHDs (six atrial septal defects, four ventricular septal defects, one persistent left superior vena cava, and one mild Ebstein anomaly). All these CHDs were difficult to detect, even after the preserved ultrasound images and movies were reexamined.

**Table 4:** False-negative cases in first-trimester screening that were detected during the second trimester

Case	Findings at second-trimester screening			Diagnosis	Factors of false negatives			
	Four-chamber view	Three-vessel trachea view	Others		CHD present in preserved images	Inappropriate PRF	Non-posterior spine	BMI
a	Normal	No V sign	PA ascending from LV, Ao ascending from RV	TGA	+	+	–	19
b	VSD	Small PA	Both great arteries arising from RV	DORV (TOF type)	–	+	–	18
c	AVSD	Ao larger than PA	Both great arteries arising from RV	DORV, AVSD, PS, right isomerism	–	–	+	18
d	Left axis deviation	Ao larger than PA	Both great arteries arising from RV	DORV (TOF type)	+	–	–	21
e	VSD	PLSVC	Both great arteries arising from RV	DORV, VSD, PLSVC, ASD	–	–	+	24
f	VSD	Normal	–	VSD	–	+	–	17
g	RA larger than LA	Reversed PA flow	–	Ebstein anomaly	–	–	+	20

*CHD* congenital heart disease, *PRF* pulse repetition frequency, *BMI* body mass index, *VSD* ventricle septal defect, *AVSD* atrioventricular septal defect, *PLSVC* persistent left superior vena cava, *TGA* transposition of great arteries, *DORV* double outlet right ventricle, *TOF* tetralogy of Fallot, *PS* pulmonary artery stenosis, *RA* right atrium, *LA* left atrium, *Ao* aorta, *PA* pulmonary artery

### Factors that may decrease the detection rate

**Fig. 3:**
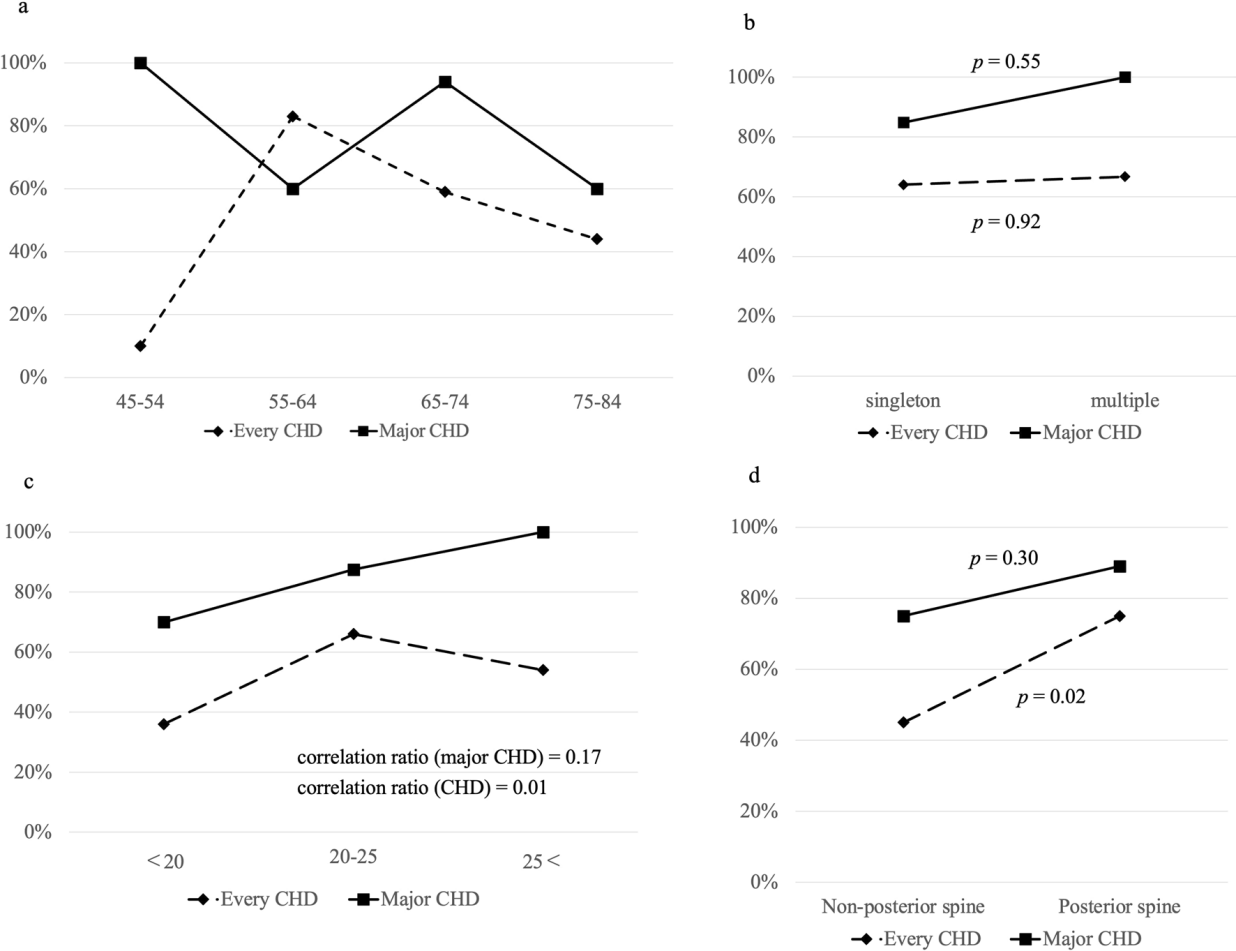
Risk factors that may decrease the detection rate. **a** Detection rate classified by fetal crown-rump length (mm). **b** Detection rate classified by number of fetuses. **c** Detection rate classified by maternal body mass index. d. Detection rate classified by position of the fetal spine. *CHD* congenital heart disease, *CRL* crown-rump length, *BMI* body mass index

There was no significant difference in the CRL detection rates (Fig. 3a) and number of fetuses (odds ratio (OR): 1.12, 95% confidence interval (CI): 0.09–13.3, Fig. 3b). There was a weak correlation between the major CHD detection rate and lower maternal BMI (correlation ratio: 0.17, Fig. 3c). The detection rate of every CHD was significantly higher when the fetuses were evaluated with their spine at the 5–7 o’clock position (posterior spine) than at the other positions (75% and 45%, OR: 3.82, 95% CI 1.16–12.5, *p* = 0.02, Fig. 3d).

## Discussion

This study aimed to elucidate the possible factors affecting the detection rates in first-trimester fetal CHD screening. Two factors significantly decreased the detection rate: the position of the fetal spine and low maternal BMI.

First, the detection rate of major CHD was 85.7%, which was higher than the results of a 2022 systematic review and meta-analysis [5] (55.80% and 67.74% in the non-high-risk and high-risk groups, respectively). This was achieved without impairing the specificity or positive predictive value. At our facility, ultrasound images are obtained or checked by a board-certified fellow of the Japan Society of Ultrasonics in Medicine with sufficient experience as a perinatologist. This double-check system is meant for quality control and trainee education, which may have had a positive effect on the high performance of CHD screening in this study. Moreover, the higher detection rate in our study may have been due to advances in ultrasound equipment in the past years. However, approximately 14% of major CHD cases were screened negative in the first trimester. All these fetuses had a well-balanced four-chamber view, even in the second trimester, and required precise evaluation of the outflow tract. In fetuses with a functional impairment of the valve, such as pulmonary stenosis or regurgitation, changes in the morphology would progress during pregnancy [6]. This poses a challenge in detecting these types of CHDs in the first trimester, which means that observing the outflow tract in the second trimester is essential for prenatal CHD diagnosis.

Two factors significantly decreased the detection rate: the position of the fetal spine and low maternal BMI. The image must be adjusted when the fetal spine is on the posterior side while evaluating the fetal heart in the first trimester [7]. When the fetal spine is anterior to the fetal heart, the acoustic shadow of the spine prevents adequate evaluation. When the fetal spine is lateral to the fetal heart, it is difficult to evaluate the color flow of the outflow tract to the three-vessel trachea view because the ultrasound beam runs vertical to the vessels. A decrease in image quality was seen in cases with a lateral spine in the literature, although a statistical difference was not reported [8]. Some previous studies only focused on the spine not being anterior to the heart [9] and underestimated the influence of the lateral fetal spine on the evaluation of the fetal heart, which may be the reason for the type 2 error. Evaluating suboptimal images is unacceptable at the first-trimester CHD screening because of the lower detection rate compared with the second trimester. Thus, ensuring that the fetal spine is posterior to the fetal heart is essential, and efforts must be made to obtain images at this position.

It is a novel finding that low maternal BMI decreases the detection rate of CHD screening in early pregnancy. High maternal BMI has been described as a factor for reduced detection rates in some previous studies [7], whereas some studies could not find a significant relationship between them [8, 10, 11]. Botteli et al. [7] reported that the risk factors for false negatives in early screening for CHD can be classified into three categories: technical, human, and acoustic windows. Maternal obesity is classified in the acoustic window category. In contrast, according to our results, low maternal BMI may join the same acoustic window category. Mothers with a lower BMI have several physical disadvantages compared to those with a higher BMI. When we scan the fetus, we use the three-dimensional maternal abdomen to optimize the images. This is particularly important in the first trimester. However, a relatively small uterus compared to that in the second or third trimester is buried deep in the pelvis, and the ilium impedes probe manipulation. This is more evident in mothers with a low BMI as there is a significant indentation between the ilium and the abdomen. In some fetal positions, it is impossible to evaluate the fetal heart in an appropriate cross-section because of the anteflexed uterus and impeded probe manipulation. Under these circumstances, we ask the patient to wait for 30 min to fill the bladder as it may dull the angle of the anteflexed uterus. Using a smaller transabdominal probe or changing to transvaginal ultrasonography provides a possibility to resolve this problem. Transvaginal ultrasonography is said to be helpful in obese patients [1, 2]. In patients with a low BMI, a transvaginal probe also could be helpful for evaluating the fetus from a different angle to obtain better images. Recognizing that a low BMI also makes it difficult to obtain an ideal image for evaluation is important, especially in the first trimester, where there is a disadvantage of relatively low-quality images.

The strength of this study is that it is one of the few to focus on the cause of decreased CHD detection rates. The low detection rate of CHD in fetuses with non-posterior spines has not been demonstrated in many previous studies. Additionally, to the best of our knowledge, low maternal BMI has never been stated as a risk factor for false negatives, although many sonographers may have experienced difficulty in probe manipulation. However, this study has several limitations. First, there is a possibility of false negatives in cases that were delivered at other facilities, which we could not monitor. Second, the average BMI in this study was lower than that reported in previous studies reporting the accuracy of first-trimester cardiac screening. This is mainly because the average body shape of East Asians differs from that of Caucasians and Black people [12], which may raise questions about external validity in different areas of the world. Particularly in the Japanese population, the average BMI is lower even compared to other East Asian countries, and our study had a similar distribution of maternal BMI compared to a previous nationwide study that took place in Japan [13]. Studies in the Japanese population are valuable considering that first-trimester CHD screening is still not popular in Japan. We hope that further studies conducted in Japan will reinforce our data.

## Conclusion

This study provides further evidence supporting the usefulness of first-trimester CHD screening. Ensuring that the images are evaluated only when the fetal spine is posterior to the heart can increase the detection rate. Additionally, it is important to recognize that low maternal BMI is a factor for false negatives due to restriction of probing for the ideal image. Some CHDs are difficult to detect in the first trimester, and a perfect diagnosis is still challenging compared to second-trimester screening.

## Data Availability

The data that support the findings of this study are available from the corresponding author upon reasonable request.
