# Correction: Factors affecting the accuracy of fetal cardiac ultrasound screening in the first trimester of pregnancy

**DOI:** 10.1007/s10396-025-01542-3

**Published:** 2025-04-15

**Authors:** Shin Hashiramoto, Mayumi Kaneko, Hiroko Takita, Yuka Yamashita, Ryu Matsuoka, Akihiko Sekizawa

**Affiliations:** Department of Obstetrics and Gynecology, Showa University Hospital, Showa University School of Medicine, 1-5-8 Hatanodai, Shinagawa-ku, Tokyo, 142-8666 Japan

**Correction: Journal of Medical Ultrasonics (2024) 52:131–138** 10.1007/s10396-024-01505-0

The article “Factors affecting the accuracy of fetal cardiac ultrasound screening in the first trimester of pregnancy”, written by Shin Hashiramoto, Mayumi Kaneko, Hiroko Takita, Yuka Yamashita, Ryu Matsuoka and Akihiko Sekizawa, was originally published under exclusive license to The Author(s), under exclusive licence to The Japan Society of Ultrasonics in Medicine. As a result of the subsequent decision to publish the article under the open access model, the article’s copyright notice was changed on 22 March 2025 to © The Author(s) 2024 and the article is now distributed under a Creative Commons Attribution 4.0 International License, which permits use, sharing, adaptation, distribution and reproduction in any medium or format, as long as you give appropriate credit to the original author(s) and the source, provide a link to the Creative Commons licence, and indicate if changes were made. The images or other third party material in this article are included in the article’s Creative Commons licence, unless indicated otherwise in a credit line to the material. If material is not included in the article’s Creative Commons licence and your intended use is not permitted by statutory regulation or exceeds the permitted use, you will need to obtain permission directly from the copyright holder. To view a copy of this licence, visit http://creativecommons.org/licenses/by/4.0.

The original article has been corrected.

